# Robotics, remote medicine, AI: Oh my!

**DOI:** 10.1016/j.hrcr.2024.01.011

**Published:** 2024-01-23

**Authors:** J. Peter Weiss

**Affiliations:** University of Arizona College of Medicine – Phoenix, Phoenix, Arizona

In this issue of *Heart Rhythm Case Reports*, Griffiths and colleagues^1^ present “Catheter ablation of premature ventricular contractions from the right anterolateral papillary muscle: A case report of a combined ECG-imaging and remote magnetic navigation approach.” This interesting case resonates on several levels, as a commonly faced clinical challenge and as the cooperative implementation of several potentially transformational technologies.

Patients with idiopathic premature ventricular contractions (PVCs) will often have an optimistic prognosis for successful and safe ablation. However, those with non–outflow tract PVCs, in particular those with papillary muscle PVCs (PM-PVCs), pose a relative challenge. There is limited data specific to right ventricular (RV) PM-PVCs, with reports finding prevalence rare at approximately 5% of ventricular ablations.^2^, ^3^, ^4^ In multicenter analysis of idiopathic PVC ablation, overall acute success was 84% and longer-term medication-free success 71%, with major complication rate of 2.4%. However, PM-PVCs were relatively rare in this dataset (again 5%) and procedures were characterized by longer times, higher fluoroscopy use, longer radiofrequency application, and lower success rates compared with all other sites of origin other than the epicardium.^5^

A first challenge is the anatomical complexity of PMs as 3-dimensional structures extending into the ventricular cavity, limiting potential for accurate visualization. Second is the high mobility of PMs, with limitations of catheter contact and stability impacting the potential for accurate mapping and effective ablation. Third is the potential for inaccurate mapping owing to the presence of anisotropic conduction and potential deep site of origin leading to changing exit sites. This case also exemplifies the challenge of a symptomatic patient with frequent PVCs on monitoring and a prior failed ablation who presents with only rare ectopy. Finally, we see the potential for remote telerobotic consultation to overcome the relative isolation within which most of us practice.

Regarding anatomic visualization, traditional electroanatomical maps (EAM) are optimized to present a 2-dimensional shell, with limited potential to accurately present intracavitary 3D structures. In this case, visualization was facilitated by magnetic resonance imaging–derived 3D reconstruction using the View Into Ventricular Onset (VIVO; Catheter Precision, Fort Mill, SC) system, with images imported into the EAM (CARTO 3; Biosense-Webster, Diamond Bar, CA). As seen in Figure 2 of the report, the PM and moderator band are discretely visualized, enhancing understanding of the anatomy in comparison to EAM alone. Several publications have highlighted the use of integrated intracardiac ultrasound (ICE) with potential for improved outcomes.^6^, ^7^, ^8^ In our lab, ICE is frequently employed for enhanced real-time visualization of structures, catheter contact, and lesion formation. In the presented case, as is common in much of the world, ICE may not be available/affordable.

Precise localization of the PVC site of origin is addressed in this case using noninvasive electrocardiogram imaging (ECGi) with VIVO to create an AI-driven patient- and PVC-specific electroanatomic “roadmap” prior to the procedure. Accuracy of this technology has been validated, with prediction of PVC and ventricular tachycardia focus accuracy of 85% and 88%, respectively.^9^ It is clear that adequate precision can be limited using EAM alone in the setting of complex anatomy, even with high-density multipolar catheters. Here, VIVO localized the PVC origin to the base of the RV anterior PM, with accuracy corroborated by pace-map match, ablation-stimulated burst of identical ectopy, and patient clinical improvement. This is contrasted with the initial attempt during which the PVC was mistakenly mapped to the RV free wall using EAM with high-density multipolar mapping alone, leading to clinical failure.

The advantage of ECGi/VIVO localization is also highlighted in the ability to treat this patient who presented with only rare ectopy, limiting ability to perform real-time activation mapping. Despite pre- and intraprocedural management to optimize PVC occurrence, there are occasions where PVCs simply do not present themselves with useful frequency. At that point, the decision is often made to abandon the procedure, with frustration resulting for all involved. In this case, anticipatory management with ECGi capture of the clinical PVC with a single recorded beat was an invaluable guide toward effective treatment.

Catheter contact and stability is a known challenge in the treatment of PM-PVCs. Characteristics of manual pull-wire catheters include translation of action from the handle to the tip through stiff components with limited curvature and directional degrees of freedom. This creates an unavoidable mismatch with the flexible, dynamic, and complex geometry of the heart.^10^ Even with contact force measurement, there remain issues with contact variability and limited validated demonstration of improved clinical outcomes.^11^^,^^12^ In contrast is the unique biophysics of remote magnetic navigation (RMN) (Stereotaxis, St. Louis, MO), characterized by a flexible catheter with an atraumatic magnetic tip navigated and pulled into consistent focal contact by a remotely controlled directional magnetic field with unlimited degrees of freedom. These attributes lead to enhanced precision, ease of catheter navigation independent of operator dexterity, and catheter stability even on highly mobile structures. Experience has included publications specific to the ablation of PM-PVCs as well as improved outcomes vs manual ablation in patients with failed prior ablation of idiopathic ventricular arrhythmias.^13^^,^^14^ In this case, RMN was successfully used to deliver significant ablation energy to the target tissues with clear physiologic effect. In our experience, RMN offers uniquely stable tissue contact when mapping and ablating PM-PVCs and has become the standard approach for several operators in our lab.

A final highlight is the introduction of remote expertise into real-time care. In the midst of an increasingly networked society, electrophysiology procedures have largely remained limited to the isolated silos of our labs. In addition to the local availability of technology and operator skill, broad clinical experience and expertise are valuable resources that we should seek to share without temporal or geographic boundaries. In this case, the providers leveraged the unique capabilities of the Odyssey Cinema component of the Stereotaxis system, which allows real-time remote visualization and even mouse control of procedural image sources through secure Health Insurance Portability and Accountability Act–compliant VPN access. Thus, this patient had the advantage of real-time in-procedure consultation with a remote expert in addition to the local physicians.

The interest of this report lies in the successful treatment of a challenging patient through a combination of advanced technologies accessible through an open electrophysiology ecosystem. It also hints at the future, which could soon include the automation of catheter navigation based on AI-informed automated mapping, potentially with remote telerobotic guidance. Each of these is a step along the essential pathway toward reduced human error and enhanced ability to optimize the care of our patients.

## Disclosures

Dr Weiss: Consultant/Speaker: Stereotaxis, Abbott, Circa Scientific, Talon Surgical.
